# Digital Twin Meets Knowledge Graph for Intelligent Manufacturing Processes

**DOI:** 10.3390/s24082618

**Published:** 2024-04-19

**Authors:** Georgia Stavropoulou, Konstantinos Tsitseklis, Lydia Mavraidi, Kuo-I Chang, Anastasios Zafeiropoulos, Vasileios Karyotis, Symeon Papavassiliou

**Affiliations:** 1 School of Electrical and Computer Engineering, National Technical University of Athens, 157 80 Athens, Greece; gstavr@netmode.ntua.gr (G.S.); ktsitseklis@netmode.ntua.gr (K.T.); lmavraidi@netmode.ntua.gr (L.M.); papavass@mail.ntua.gr (S.P.); 2 Fraunhofer Institute for Mechanics of Materials IWM, 79108 Freiburg, Germany; kuo-i.chang@iwm.fraunhofer.de; 3 Department of Informatics, Ionian University, 491 00 Corfu, Greece; karyotis@ionio.gr

**Keywords:** digital twins, knowledge graphs, material ontologies, material manufacturing, laser glass bending

## Abstract

In the highly competitive field of material manufacturing, stakeholders strive for the increased quality of the end products, reduced cost of operation, and the timely completion of their business processes. Digital twin (DT) technologies are considered major enablers that can be deployed to assist the development and effective provision of manufacturing processes. Additionally, knowledge graphs (KG) have emerged as efficient tools in the industrial domain and are able to efficiently represent data from various disciplines in a structured manner while also supporting advanced analytics. This paper proposes a solution that integrates a KG and DTs. Through this synergy, we aimed to develop highly autonomous and flexible DTs that utilize the semantic knowledge stored in the KG to better support advanced functionalities. The developed KG stores information about materials and their properties and details about the processes in which they are involved, following a flexible schema that is not domain specific. The DT comprises smaller Virtual Objects (VOs), each one acting as an abstraction of a single step of the Industrial Business Process (IBP), providing the necessary functionalities that simulate the corresponding real-world process. By executing appropriate queries to the KG, the DT can orchestrate the operation of the VOs and their physical counterparts and configure their parameters accordingly, in this way increasing its self-awareness. In this article, the architecture of such a solution is presented and its application in a real laser glass bending process is showcased.

## 1. Introduction

In today’s highly volatile industrial environment, businesses strive for the continuous integration of innovations that will allow for cost reduction, reduced time to market, and the minimization of errors that oftentimes occur during production due to faulty equipment or substandard configurations input by inexperienced personnel. In this landscape, smart and automated manufacturing has emerged as one of the pillars for achieving these goals [1]. Advancements in the fields of electronics have made the existence of powerful devices possible (e.g., Raspberry Pis, ESP boards, etc.), near the network edge. These devices coupled with the abilities of Internet-of-Things (IoT) devices (e.g., sensors, actuators, etc.) allow for the development of solutions that enable the design of intelligent manufacturing models [2]. These models are beneficial for enterprises because they provide means for optimizing workflows and enable better monitoring.

In addition to these developments, semantics play a crucial role in material manufacturing since they provide a means to organize vast amounts of heterogeneous data about materials and their properties, the structure of industrial business processes, and the employed devices in a structured manner [3,4]. Knowledge graphs emerge as suitable tools to represent data that follow ontologies. They provide a means to handle and analyze data originating from different aspects of the operation of a business. They support advanced information retrieval and visualizations, and their use offers better understanding of the domain, aiding in designing more robust intelligent manufacturing process tools.

Digital twins have been proposed for handling numerous aspects of the manufacturing processes offering capabilities such as predictive maintenance, real-time monitoring, and self-awareness [5]. Despite being in the spotlight for the past years as an innovative tool, they come with certain important shortcomings. First of all, the vast majority of solutions constitute proprietary software [6], which can pose a barrier for their adoption by the numerous Small-Medium Enterprises (SMEs) active in the manufacturing domain. The work in question only considers open-source tools, showcasing their flexibility to address real scenarios in the manufacturing domain.

Moreover, DTs are highly specific to their domain and require significant effort in their configuration to model the materials and devices used by the physical counterpart (i.e., the actual equipment) [6] and their dependencies. Combining DTs with a KG can mitigate this need, since it adds a semantic layer allowing for the integration of complex relationships between different components of the digital twin. It allows, in this way, a more efficient orchestration enforced by the digital twin in the physical and software assets it emulates. The present work, focusing on the material manufacturing domain, adopts this approach and proposes the connection of the DT to a KG that has a dual focus. This dual focus allows the modeling of both materials and properties and of the equipment used for each step of the manufacturing process.

In this article, a solution for designing intelligent manufacturing processes is presented, based on the concept that a knowledge graph is accessible by a digital twin, and therefore, additional context can be developed. The employed components were both designed for manufacturing processes. The specific contributions of the aforementioned work are the following:

- A novel KG schema was designed, focusing on two aspects. The first aims at providing holistic descriptions of the materials used and their properties. The second aspect aims at describing the industrial business processes (IBP) during which the materials are transformed into the end products. The KG is stored as a neo4j database;
- By using the Nephele VO software stack [7,8], a novel architecture for designing the digital twin (DT) is proposed. Based on this software stack, a Virtual Object (VO) is considered as a virtual counterpart of an IoT device that can be deployed at the edge part of the infrastructure. By combining various VOs, a DT can be implemented, acting as an aggregating point that can fuse knowledge and exploit functions provided by the VOs. This DT is capable of orchestrating, configuring parameters, and detecting faults in the manufacturing operation with the aid of the KG through suitable queries;
- The ways in which the adoption of the proposed solution can be beneficial for businesses in the manufacturing domain are discussed. The combination of a KG and DTs allows increased self-awareness of the DT components by capitulating on the semantics stored in the KG. Moreover, the DT, by accessing the stored information about the devices and the disposition conditions, can more effectively achieve tasks such as predictive maintenance;
- Finally, the ways in which the solution can be used for a real-world use case, namely, a glass bending operation that is achieved by using a furnace and a laser device, are presented. Both the mapping of the components (KGs and VOs) for this particular process and the implementation of two basic functionalities are showcased in detail.

The remainder of this paper is structured as follows. Section 2 discusses related works on manufacturing ontologies, knowledge bases, and their application in the context of digital twins. In Section 3, the knowledge graph schema is presented and the proposed digital twin architecture is discussed in detail. Then, Section 4 briefly presents how including the knowledge graph enhances various operations of the digital twin. Afterwards, Section 5 showcases the application of the proposed solution in a real-world scenario concerning a laser glass bending process. In Section 6, insightful observations about the advantages of the proposed approach and the current challenges and limitations are presented. Finally, Section 7 concludes the paper.

## 2. Related Work

Ontologies have been used extensively for specifying a concept regarding specific domain information and relevant processing methods. They provide a clear and formal description of a shared understanding of a domain [4]. Ontologies serve as data models for domain concepts using terms like classes (entities) and relationships (properties) [3]. When an ontology is populated with instances, meaning specific entities or examples that belong to concepts defined by the ontology, it forms a knowledge base, also known as a knowledge graph [3]. Advancements in technology and the increasing presence of various field devices like sensors, embedded systems, and autonomous robots have significantly boosted Industry 4.0 (I4.0) manufacturing processes. These diverse field devices interact in real time, generating substantial amounts of valuable data throughout production. However, the heterogeneous nature of these devices, the diversity of their data outputs, and their varying degrees of interoperability pose challenges for maximizing the efficiency of I4.0 industrial operations. Ontologies have emerged as crucial tools for modeling device concepts, capabilities, parameters, processes, etc., within the context of I4.0, facilitating integration and interoperability efforts [3], addressing a range of issues, including domain knowledge modeling, integration of the Internet of Things (IoT), and more [3]. This work addresses the above matters by creating a knowledge graph structure with a dual focus. The first point of interest is the broad descriptions of the materials used, along with their properties. The second area pertains to detailing the industrial business processes (IBP) involved in altering these materials into final products. Several initiatives aim at encapsulating the domain knowledge pertinent to I4.0 through modular ontologies to fulfill manufacturing production needs, e.g., Process Specification Language (PSL), ONTOlogy for Product Data Management (ONTO-PDM), MAnufacturing Semantic ONtology (MASON), ADAptive holonic COntrol aRchitecture (ADACOR), etc. [3,4]. BPMN, or the Business Process Model and Notation, stands as a widely accepted industry standard that is also utilized for the modeling of business processes. In [9], extensions to the BPMN designed specifically for process modeling within the manufacturing domain are introduced, with a particular focus on production processes.

I4.0 has been founded on enablers such as big data, cloud computing, and IoT. These technologies also form the foundation for a novel simulation method, which capitalizes on the widespread connectivity in production systems to provide real-time synchronization with the production site. Such innovative simulation methods are referred to as digital twins (DTs) [10], serving as a virtual and computerized replica of a physical system [11,12]. Digital twins have the capability to choose from a range of actions aiming at coordinating and executing the entire production system in an optimal manner [11]. Through simulation, prediction, and optimization of physical systems and processes, the DT contributes towards the achievement of various objectives [11,12]. DTs enable the real-time monitoring and control of devices and cyber-physical production components across network infrastructures, facilitating a more seamless integration and synchronization between the physical and virtual realms [11]. A DT requires three essential elements: an information model that summarizes the characteristics of a physical object, a communication system facilitating two-way data exchange between the DT and its physical counterpart, and a data processing module capable of extracting insights from diverse data sources to create a dynamic representation of the physical object [12].

DTs can be categorized into three distinct classes based on their level of integration. Initially, the Digital Model (DM) represents a physical object, either existing or planned, without utilizing automated data exchange between the physical and digital entities. The Digital Shadow (DS) incorporates automated one-way data transfer from an existing physical object to its digital counterpart. Changes in the physical object’s state result in corresponding alterations in the digital representation, but not vice versa. Lastly, the DT encompasses full bidirectional data exchange between the physical and digital entities. While there is limited literature on the most advanced developmental stage, i.e., the DT, there is relatively more information on the DM and DS [11]. The work presented in this article implements the most advanced level of integration, meaning the digital twin, since it supports communication with the physical counterpart and provides a means for the data analysis of the generated data originating from IoT devices. DT-driven Predictive Health Maintenance (PHM) integrates physical and virtual data, real-time and historical data, and data fusion, aligning with the trend of smart manufacturing driven by big data [13,14]. These applications primarily focus on monitoring functions (such as status monitoring and process visualization) and prediction functions (like fault prognosis, product life-cycle management, and process optimization), serving as decision-making support tools for humans [12]. In [15], a distributed DT structure designed to enhance decision-making regarding abnormal occurrences at the local level is introduced. The local decision-making module utilizes an adaptive threshold approach and the effectiveness of the framework is demonstrated on an Industry 4.0 pilot line. Our proposed solution conforms with [15] in terms of the monitoring and predictive maintenance capabilities of the DTs and goes a step beyond by exploiting knowledge graphs, thus enabling dynamic DT configuration and orchestration.

By definition, DTs rely on current and coherent data, information, and knowledge. They can also be regarded as models themselves, applicable across various contexts within production systems. The authors in [16] shed light on the significance of integrating expert knowledge, data analytics, and Knowledge-Based Systems (KBSs) for production systems in general and the development of DTs specifically. KBSs, like knowledge graphs, play a crucial role in the realm of DTs by offering explicit semantics, such as representations of DTs using web ontology language (OWL) [16]. In the present study, the knowledge graph introduces a semantic layer that allows better monitoring of the business process and enables coordination among various elements of the digital twin. The primary application of knowledge graphs is knowledge fusion [17]. Ontologies and knowledge graphs offer solutions to enhance DTs with advanced cognitive capabilities. Cognitive Digital Twin (CDT) models dealing with diverse data, information, and knowledge from complex systems across various domains and lifecycle stages pose challenges for alignment among different DTs and stakeholders. The aforementioned semantic technologies address this issue by establishing meaningful connections among heterogeneous data sources. CDTs are able to optimize procedures and make decisions via marshaling the support of knowledge graph modeling and reasoning. These also serve as the foundation for key functional layers including data ingestion and processing, model management, service management, and twin management [18]. The study in [19] introduces an ontology model and a KG designed to amalgamate simple DTs into a more intricate DT, which encompasses functionalities like information fusion, multi-scale association, and multi-context interaction. The ontology provides a comprehensive repository of information for entities across diverse DTs, while the KG helps in establishing structural connections among DTs of different scales. The DT developed in this work not only manages and orchestrates VOs to simulate manufacturing processes, adapting dynamically to changes in steps or sequences without manual intervention, but is also able to set operation parameters in the physical counterpart and produce alerts, making use of the data stored in the KG.

In [20], the authors propose a method to extract valuable insights from vast amounts of production line data and improve manufacturing process management through enhanced reasoning abilities. They introduce a pipeline that automates the extraction of semantic relationships from sensor data, consisting of four key stages: feature extraction, ontology-based manipulation, knowledge graph generation, and relation inference. The research in [21] argues that DTs must possess the capacity to encompass the attributes of an asset as delineated by the manufacturer, its real-time state, and its interactions within intricate systems. It states that semantic technologies, particularly ontologies, offer a promising modeling approach for digital twins and are supported by readily available tools for designing, managing, querying, and exploring semantic models. The present paper introduces an ontology that models IBPs and the exploited materials in detail and, furthermore, capitalizes on this valuable knowledge to improve the decision-making processes. The authors of [22] introduce a method for creating actionable cognitive twins tailored to demand forecasting and production planning within manufacturing facilities, utilizing a knowledge graph approach. This approach enables the semantic representation of industrial processes, encompassing data identification and the simulation and utilization of AI algorithms for forecasting purposes. In [23], an ontology is proposed to model a shop-floor DT, covering assets, actors, data sources, algorithms (especially AI), and decision-making processes. This ontology enhances DTs with cognitive features and creates a KG to provide context to data and algorithm outcomes, improving decision-making. The system learns from historical data to detect anomalies and trigger contextual actions. It also uses probabilistic machine learning (ML) and heuristics to aid in production scheduling [23]. Our study improves the aforementioned approach by also representing the materials used in IBP and their critical states, enabling the possibility of the DT to create alerts, configure itself, and organize its individual components. The research in [24] introduces an ontology for digital twin modeling capable of graphical visualization of digital twins, enabling both device mapping and customizable visual attribute representation. A case study validated the ontology’s effectiveness in mapping physical devices and facilitating DT development. The authors in [25] explain the concept of Cognitive Twins (CT) and propose a framework centered around KGs, which helps to identify and manage the structures of virtual model assets to facilitate CT development. In [26], the authors define and demonstrate DTs in an industrial context using KGs. They propose a methodology for creating DTs from different semantic viewpoints, one centered on production parameters and the other on data flow issues. In [27], a framework for a Knowledge-Powered Digital Twin Manufacturing Cell (KDTMC) is presented, aiming to facilitate intelligent manufacturing. It utilizes DT models, dynamic knowledge bases, and knowledge-based intelligent skills to enable autonomous manufacturing through sensing, simulating, comprehending, forecasting, optimizing, and managing processes.

Historical equipment monitoring data are used to derive features that indicate equipment health, forming the basis for data-driven predictive and diagnostic models [28]. In [29], various ML algorithms are discussed for predictive maintenance in I4.0, highlighting their respective advantages. Similarly, Ref. [30] offers a comprehensive review of the existing literature on the topic, identifying anomaly detection and fault isolation as classification or clustering challenges, and prognostics as a regression-related issue. The DT examined in the present work is capable of incorporating all former functionalities and enhancing them by exploiting information stored in the KG. The authors in [31] classify the current AI-driven methods for smart manufacturing and predictive maintenance into four categories: (i) data-centric methods; (ii) methods based on physical models; (iii) knowledge-driven approaches; and (iv) hybrid model-based methods. In [32], it is argued that logistic regression (LR) outperforms artificial neural networks (ANN) and support vector machines (SVM) in the binary classification problem of machine health due to its parametric structure utilizing explanatory variables, which offers a deeper understanding of how changes in input variables affect the probability classification [32,33]. The hybrid approach in [31] uses statistical AI technologies such as ML and chronicle mining (a special type of sequential pattern mining approach) to extract machine degradation models from industrial data. In [34], a data-driven approach is adopted using a nonlinear auto-regressive neural network with an external (exogenous) input (NARX) to predict the dynamic model of the physical systems and then build a stochastic model predictive controller (MPC). In [35], a hybrid method is used, combining data-driven prediction (an RBF neural network algorithm) and model-based prediction (a particle filter algorithm, which leverages real-time data to continuously update the current state estimation, thereby enhancing prediction accuracy).

## 3. Proposed Architecture

In this section, details on the architecture of the proposed solution of combining KGs and DTs are presented. First, the KG’s schema is showcased, explaining its various entity types and relationships and highlighting the most crucial ones for the connection to the DT. Next, the DT architecture is provided, explaining how smaller components interact in order to produce a DT. Elements of the Nephele VO stack are used and their purpose in generating highly flexible twins that can support a number of functionalities is highlighted. Finally, the overall architecture is discussed, explaining how the two software components interact.

### 3.1. Knowledge Graph Schema

**Figure 1:**
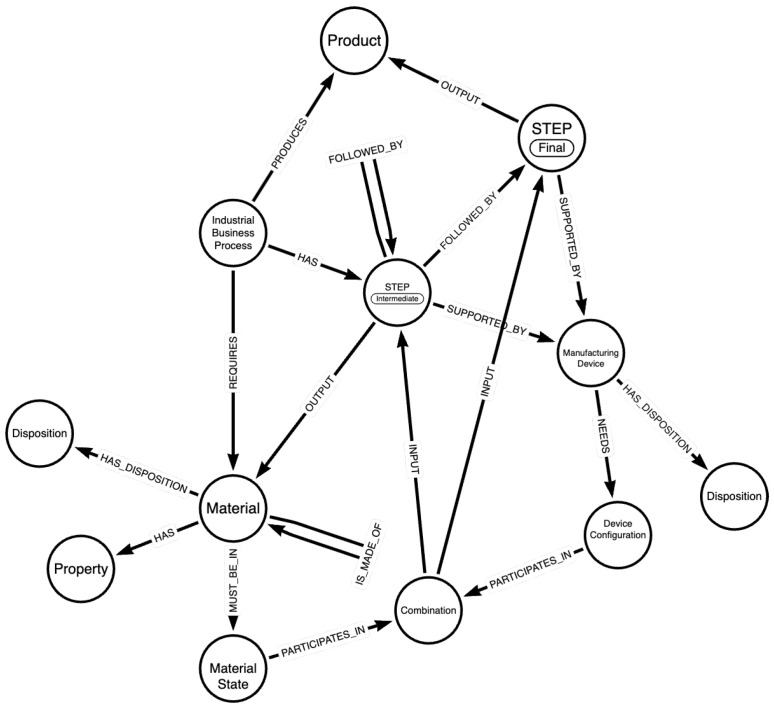
Knowledge graph schema.

The structure of the KG is described by a knowledge graph schema, provided in Figure 1. This schema illustrates the steps describing the considered industrial processes, along with the materials and devices involved in each step, to produce a certain product that complies with specific standards. The main entities comprising the provided schema along with a description of the considered relationships with other entities are as follows:

- ***Industrial Business Process***: The manufacturing process that the schema aims to describe as comprehensively as possible. It “HAS” steps (Intermediate and Final) that involve certain materials (relationship “REQUIRES”) and devices that need to be configured in a specific way to produce the desired material to be processed in the next step. The final step of the process outputs the desired product of the industrial business process, with which it is directly linked (“PRODUCES” relationship);
- ***Step***: A step of a process describes a specific part of the manufacturing procedure and can either be Intermediate or Final. It can be followed by other steps (if the step is Intermediate), it relates to materials and is “SUPPORTED_BY” Manufacturing Devices. Depending on whether the step is Intermediate or Final, it “OUTPUTS” a material for the next step, or the final product of the process. The distinction between Intermediate and Final steps aids in traceability, allowing the end product of an IBP to be traced back to the materials and the devices employed in the process as well as their respective states and configurations;
- ***Material***: This entity describes the materials that are linked with the industrial business process. A material involved in a certain step of the process “HAS” certain ***Properties*** and “MUST_BE_IN” a specific ***Material State*** to be processed. Another entity, called ***Disposition***, relates to undesired states in which a material can be found and can lead to products with defects;
- ***Manufacturing Device***: A certain number of Manufacturing Devices are involved in the execution of a manufacturing process. They support steps of the process and handle certain materials. A device “NEEDS” a specific ***Device Configuration*** to properly process a material and relates to the ***Disposition*** entity, which describes unfavorable conditions and configurations of the devices that need to be avoided for the successful and safe implementation of the process.
- ***Combination***: At a specific step of the process, the ***Material State*** and the corresponding ***Device Configuration***, must form a combination (relationship “PARTICIPATES_IN”) that is essential for processing the material effectively. This combination characterizes each step of the process.

The *Disposition* entity is used as described in the Industrial Data Ontology, while other entities and relationships can be mapped to this ontology, the draft publication of which can be found in [36].

The schema presented above serves the purpose of storing and organizing information necessary for the integration with the digital twin framework, with a primary focus on forming recipes to contribute to the efficient and safe execution of an industrial process. However, this schema could also be leveraged for more advanced functionalities, such as data analysis, machine learning algorithms, and retrieval of similarities between materials and processes.

### 3.2. Digital Twin Architecture

The software employed for creating the DTs is the Nephele Virtual Object software stack [7]. This lightweight software stack is compliant with the W3C Web-of-Things (WoT) standard [37] and allows the virtualization of Internet-of-Things (IoT) devices in a device-independent way.

The basic components of the software stack employed in this work for designing the DTs are Virtual Objects (VOs) and composite Virtual Objects (cVOs). A Virtual Object can be seen as an abstraction of specific IoT devices such as sensors, actuators, etc. These sensors can be installed in appropriate manufacturing equipment, offering in this way the capability of virtualizing their operation. Each VO is described by its respective thing descriptor, a JSON file describing the properties, the actions, and the events of the corresponding device and offering necessary metadata that give more context to the user. The actions are implemented as separate Python scripts. More functionalities can also be implemented in Python. The defined actions and any additional functionalities are referred to as virtual functions (VFs). Composite VOs are software entities that can combine information originating from multiple VOs offering in this way more advanced functionalities and a point of management of the VOs, orchestrating their collaboration towards emulating/simulating a complex physical counterpart (i.e., the physical twin), while also making real-time monitoring and process control feasible. The functionalities of the cVO (or DT) can use the results obtained from the VFs deployed in the simple VOs that act as its parts as inputs. Data generated by the IoT devices are stored in a time-series database, accessible from the actions through Python libraries.

Virtual objects, VOs or cVOs, can “communicate” with each other either through HTTP, MQTT, or coAP. In this work, it is considered that the communication takes place over HTTP APIs. These details are specified for each VO inside the respective yaml descriptor file.

Communication through the HTTP API between VOs is essential for building the DT. In more technical detail, each VO exposes its properties and actions/virtual functions described in the thing descriptor file. In this manner, a (c)VO can gain access to the properties stored in another or invoke a subset of the virtual functions (i.e., those exposed as “actions”). In this way, a cVO can check the state of a VO and issue a command if necessary. For example, a cVO corresponding to a large office space can check the properties of a VO corresponding to the meeting room and, if the properties suggest that the room is empty (e.g., light but no sound), invoke the responsible action for shutting down the lights. Of course, communication is not only possible between a VO and a c(VO), but both VO-VO communication and cVO-cVO communication is feasible. The developers designing each solution need to make decisions on the manner in which the (c)VOs communicate based on how different parts of a process or a large machine cooperate.

**Figure 2:**
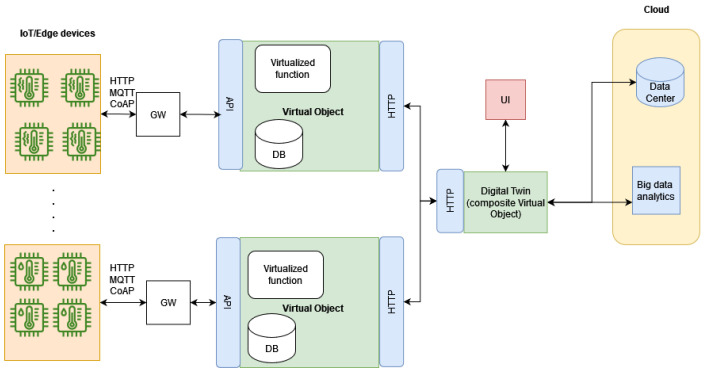
General DT architecture overview.

Based on this software, an architecture that is capable of producing the DTs for manufacturing processes is developed. In the following figure (Figure 2), the general architecture can be seen. It is essentially a three-layer architecture, including edge and cloud layers alongside the deployment layer of the toolkit. The DT toolkit can be deployed either in servers or locally at the user’s side if the computational demand of the desired functionalities is moderate.

At the edge, IoT devices are connected to the machinery and transmit data either at regular intervals or when certain conditions, set by the user, are met. These data are transmitted and can be aggregated, if necessary, at a gateway node. The VO obtains the data through a suitably defined API that can be either coAP, HTTP, or MQTT. These data can be stored in the time-series database. Virtual functions, implementing the actions defined in the things descriptors and any additional logic needed for simulating the operations handled by the physical counterpart, are implemented and executed in the same place: where the Nephele VO software stack is deployed. For operations requiring a lot of computing resources, access to cloud services can be supported as shown in the “cloud” part of the architecture. Finally, a User Interface (UI) can be developed that allows the user to access the information stored in the digital twin (i.e., view the status of the physical counterpart, ask for certain actions, perform time-series analysis, etc.).

### 3.3. Connecting the KG with the DT

**Figure 3:**
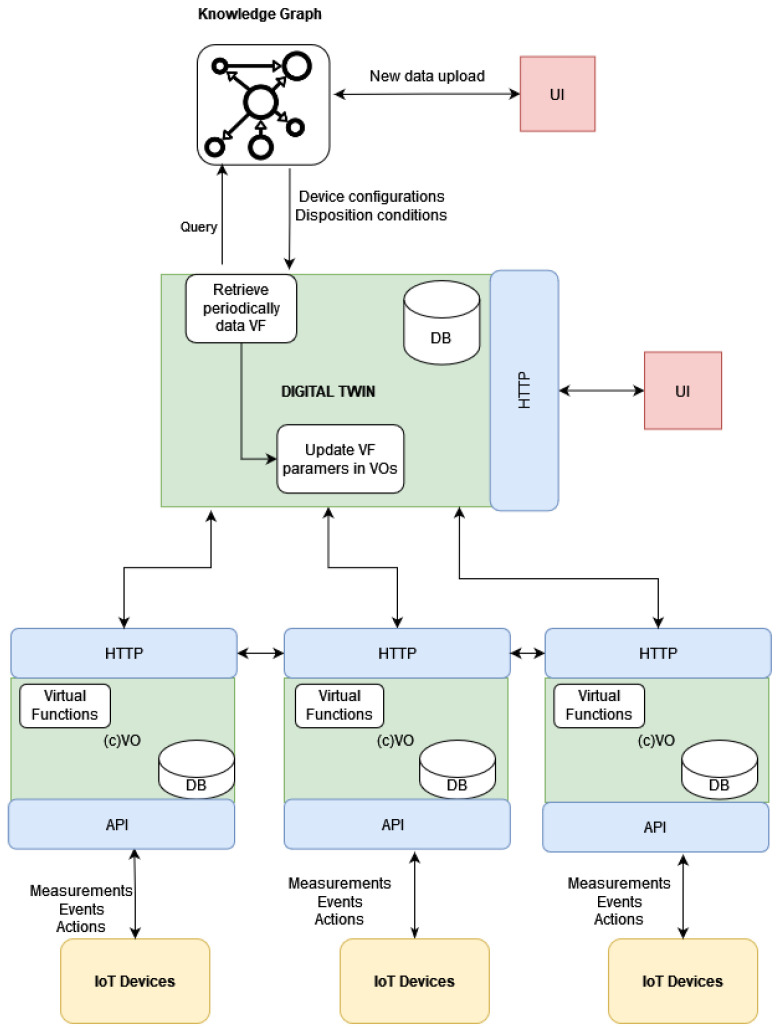
Proposed architecture schema.

The overall architecture can be seen in Figure 3. In this figure, the digital twin is considered to be comprised of multiple (composite) Virtual Objects that act as the abstractions of certain elements involved in the IBP (devices, materials, products, etc.). The developed digital twin contains, beside other functions, a specific virtual function responsible for polling data from the KG through appropriately designed queries, executed at regular intervals. If changes in certain types of nodes (i.e., the disposition of devices or materials, material states, or device configurations) are detected, these changes should be reflected in the corresponding virtual function parameters that now need to be updated. The digital twin, coordinating the operation of the rest of the VOs, notifies the subset of the VOs that needs to update these parameters. The communication between the DT and the neo4j database, where the KG is stored, is accomplished via neo4j’s Python API.

## 4. KG Enhanced Virtual Functions for the DT

### 4.1. Enhancing Self-Awareness of Digital Twin

Leveraging the information stored in the knowledge graph database, the DT can further enhance its self-awareness capabilities by accessing the stored information when required. The dual focus of the KG presented in Section 3.1 allows for storing the necessary details describing how each IBP is carried out depending on different input materials and on different products.

In greater detail, one of the key functionalities offered by the toolkit is monitoring the IBP. Using measurements originating from IoT devices embedded in the equipment, virtual functions that perform checks and produce alerts, or other forms of notifications, if the specified conditions are not met, can be implemented. These conditions oftentimes are not the same for every material employed by the user, but rather vary based on their type, composition, etc. This means that machinery may require a different configuration per material or per the specifications of the end product or for different steps of the process. Instead of developing different VOs with slightly different virtual functions, or requesting large amounts of information as the input from the user each time the DT is used, the knowledge graph offers an endpoint that the DT can access and from which it can retrieve all this information using minimum input by the user. The relative information is stored in the KG in the *Device Configuration* and *Material State* nodes for each *Step* of the process.

Besides setting or modifying the right parameters for the VOs, because of the structured manner in which the data are stored in the schema shown in Section 3.1, the order in which the steps are executed and the devices employed alongside their specific configurations are known. This allows the DT to orchestrate the operation of the VOs in order to emulate the manufacturing process. In this way, when a new step is introduced, or an existing step is modified, the DT is able to reorganize the operation of the VOs without the need of a developer to explicitly set the order in which each one should operate.

Moving one step further, if the necessary actuators are installed in the user’s site, the DT can utilize the information stored in the KG in order to explicitly set the operation parameters in the physical counterpart, or make the necessary adjustments if deviations from the described process are detected. Moreover, the *Disposition* nodes, both for materials and for the devices used in the IBP, offer information about undesired states. Using these values, the VOs can produce alerts when needed, notifying the responsible party to take action.

The enhanced self-configuration offered by the connection of the KG and the developed digital twin also allows for reducing the amount of defective products that lead to increased costs for the business and increased delivery time to the client.

### 4.2. Indicative Usage Scenarios

Following the presentation of the overall architectural approach of the proposed solution, in this section, the applicability of the solution for serving specific industrial processes, such as predictive maintenance processes and supply chain optimization, is detailed. In a predictive maintenance scenario, the DT can provide virtual replicas of physical assets of the Industry4.0 infrastructure (i.e., VOs) and enable the real-time monitoring, emulation, or simulation of such assets. Time-series data can be stored in the InfluxDB instance of the VO. Python offers numerous options for time-series analysis algorithms, such as ARIMA models, Prophet, or even machine learning algorithms like Random Forest Classifiers (RFF). The results of these techniques can be applied to identify anomalies that can be attributed to the misbehavior of the Industry4.0 infrastructure, leading to alerts for the potential maintenance of the considered devices. Such anomalies can be further justified taking into account the modeling of the overall IBP in the KG. Cascading effects through the various steps of an IBP can be also analyzed, including the risk assessment of a misbehavior in the overall IBP. Similarly, the proposed solution can also be applied for supply chain optimization within an Industry 4.0 environment. Through the development of DTs and the exploitation of the structure knowledge for IBPs in the KG, enterprises can identify bottlenecks, streamline workflows, and improve the overall supply chain performance. Finally, DTs can also be adopted for the design of products and the simulation of their behavior, considering different conditions in the IBP, as provided through the KG. In this way, iterations on the design of the products can be easily provided, reducing the time and the cost that is associated with physical prototyping.

Although, in this article, the use case presented is related to the glass industry (laser glass bending), the proposed solution is highly flexible and can be mapped to any manufacturing process. For example, it can be used in filament extrusion, which is a polymer-related process whose objective is to turn pellets of polymers into filaments suitable for activities such as 3D printing, yarn manufacturing, etc. In this process, numerous devices are involved, whose configurations highly depend on the input material, as different polymers have different properties. In such a case, the deployed VOs correspond to the devices used (e.g., extruder, godets, etc.) The KG stores information about the order of the steps and the different configurations needed for each device per input material (e.g., extruder speed, godet speed, etc.). The deployed cVO that aggregates and coordinates the operation is responsible for synchronizing the operation by setting the desired parameters in the VOs in time, while monitoring the operation and ensuring the smooth completion of the process. The VOs, through communication with the actual devices, monitor the operation of their respective equipment and, if necessary, issue commands when required (i.e., begin operation, pause, terminate, etc.).

## 5. Proof of Concept: Laser Glass Bending Process

In this section, the way in which the proposed architecture can be used in an actual manufacturing environment, i.e., a laser glass bending process, is described.

Fraunhofer IWM developed a laser glass bending process to shape glass precisely without affecting the quality of the flat areas of the glass sheet [38]. Additionally, this method enables highly customized bends with small radii, creating unique shapes, which are impossible to achieve with traditional methods.

**Figure 4:**
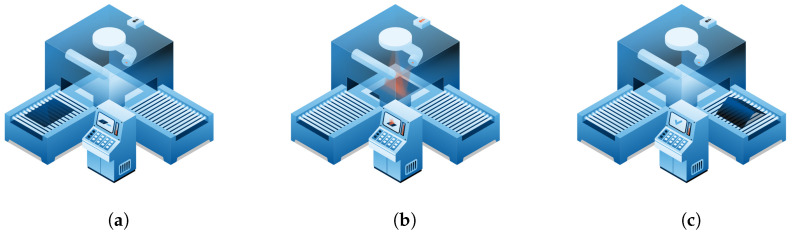
Schema of laser glass bending process. (**a**) Preheating; (**b**) localized heating and shaping; (**c**) quality check.

The process starts with preheating flat glass sheets in a hot furnace to the process target temperature. This step is monitored by thermal couples for the furnace and a thermal camera for the glass itself. Once the target temperature is reached, a high-powered CO_2_ laser beam is used to heat designated areas of the glass along the desired bend lines. The laser scanner precisely controls the number of laser cycles, path, speed, and power based on specific programs for the final product. This localized laser further heats the glass by an additional 150–200 °C and the softened section of the glass is then shaped through controlled mechanical force or gravity. Finally, the shaped glass is removed from the furnace and undergoes quality checks to ensure the bending radius and angles meet the specifications. A schematic representation of this process is shown in Figure 4.

### 5.1. Knowledge Graph Formation for This Use Case

The knowledge graph of the proposed solution stores and organizes the information of the laser glass bending process along with the steps, the types of glass (materials), and the device settings (device configurations), described at the beginning of Section 5, which are utilized to produce final glass products. For this specific industrial process, there are several steps that involve configuring the relevant devices to specific settings that are the same for each type of glass.

The devices and the elements linked to them (configurations and disposition), which are the same regardless of the type of glass they process (only the laser scanner configuration varies), are described in Table 1. The basic devices are the furnace, which is the device in which the operation takes place, the motor, which is used for moving the sheet of glass inside and outside of the furnace, and the laser, which bends the glass in the desired position.

Next, in Table 2, the steps, which are the same regardless of the material, and the devices that support them, are described in order of occurrence. The *Device Configuration* of the furnace changes depending on the step (100% for steps “Preheating furnace”, “Glass enters furnace”, and “Heating glass (global)”, 60% for “Stabilizing temperature” and “Lasering glass (local)”, and 0% for “Glass exits furnace”).

The materials that this industrial process handles participate in the Heating glass (global) *Step* and the Lasering glass (local) *Step* and produce certain products for which the *Device Configuration* (laser program) of the laser scanner *Device* that participates in the Lasering glass (local) *Step* varies. An overview is presented in Table 3. The manufactured products cover various needs, ranging from automotive glass to glass for windows with certain properties (e.g., double glazing for insulation, consisting of two-layered glass). In the latter case, only one sheet of glass is treated at a time.

**Figure 5:**
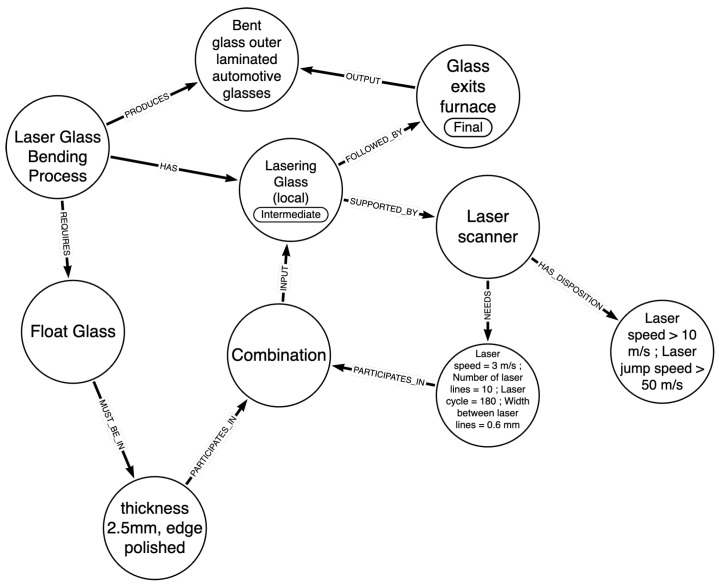
Laser glass bending process.

In Figure 5, an instance of the knowledge graph that differentiates among the different materials is provided. The instance illustrates the first Float Glass Material Lasering glass (local) step, only showing the relationship with the laser scanner and the related product. The rest of the information described in this section is not depicted in the figure, so that the representation is meaningful and not extensive. It is also worth noting that the KG schema described in Section 3.1 is a more comprehensive schema, capable of describing more complex industrial business processes than the current one, which requires fewer entities and relationships to be adequately represented. In particular, for the laser glass bending process, there is no *Disposition* and *Property* entity associated with each material and it is not regarded necessary for this process to report the output material of each step and, hence, the IS_MADE_OF relationship of the materials is not required.

### 5.2. Digital Twin Architecture

The digital twin for this specific use case is designed as follows. A composite VO monitors the entire operation and communicates with the KG by utilizing the neo4j Python library.

The database used by the composite VO contains a table matching the devices found in the graph to the corresponding VOs. This table should be provided by the user and is essential for the software since it allows the orchestration of the rest of the Virtual Objects.

The VOs that need to be defined here are the following: the furnace, motor, and laser. These are the active devices used for this process. The passive devices used for this business process, meaning devices that measure the temperature, power, etc., are considered as parts of the furnace VO, which also stores their desired configurations (coordinates, operation ranges, polling rates, etc.). All the VOs share a set of common virtual functions, but they also contain distinct ones that are tailored to the specific functionalities of their respective devices. In greater detail, all the VOs have functions that handle the data population to a time-series database. Moreover, all have an update function that is executed periodically and sets the current state of the VO. In addition, a virtual function checking the current state is responsible for notifying the composite VO on whether the device has reached the desired state in order to proceed with the next step. In this use case, the distinct functionalities for the furnace VO focus on providing visualizations, details of which are provided in Section 5.3.1. For the laser VO, the distinct functionality is setting the correct laser program, which is transmitted to the manufacturer’s site as an SLD formatted file.

The user needs to specify the type of material and end-product desired. Then, the DT toolkit accesses the neo4j database and retrieves the steps of the process together with the necessary information about the device configurations from the knowledge graph. Then, each VO monitors the respective equipment by polling the corresponding IoT devices (thermal couples, camera, etc.). When the necessary conditions for each step are met, only then is the operation allowed to continue to the next step. If some conditions are not met, then the user is notified by messages on the screen and/or emails. For example, if the temperature of the furnace does not reach (or exceeds) the specified 500 degrees (the device configuration of the furnace device for the preheating step), then it is not possible to continue to the next step successfully.

If suitable IoT equipment is installed in the manufacturing site, the digital twin can control the operation of the equipment by adjusting the power of the equipment, the position of the motor, or by controlling any other equipment that is connected.

The connection with the KG allows for increased flexibility for the laser-controlled glass bending process. A new glass type or desired product can be inserted without the need to manually set parameters in the VOs. New steps can be inserted in the knowledge graph and, provided that the table matching devices connected to the VOs is updated to include any new devices, there is no further need for modifications in the digital twin for the co-ordination of activities.

**Figure 6:**
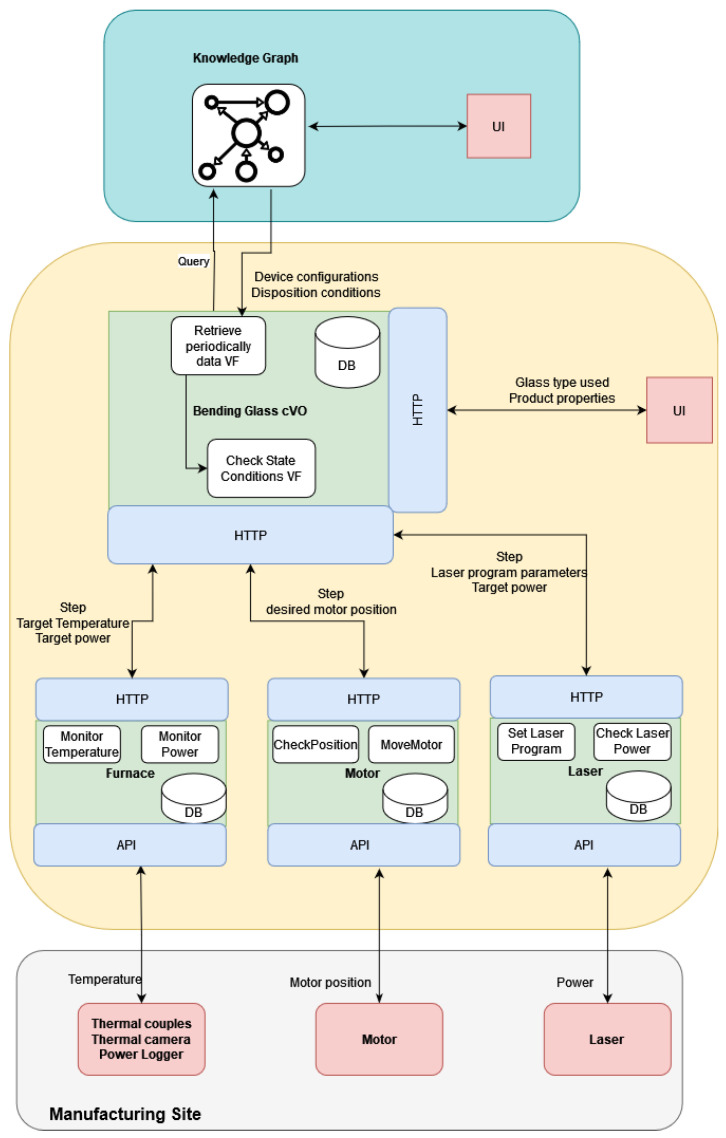
Laser glass bending process architecture.

The architecture of the DT for this particular use case can be seen in Figure 6.

### 5.3. Basic Functionalities

In this subsection, some basic functionalities of the developed digital twin are presented. All software employed is open-source and the setup in which the knowledge graph and the Virtual Objects were deployed was a laptop with AMD Ryzen 7 3.2 GHz with 16 GB RAM and 4 GB of GPU memory. The front-end used for all the screens included in the figures was implemented in Python with the NiceGUI library. For the KG, neo4j 5.12.0 was employed. For storing the time-series measurements (i.e., the temperature, power, and motor position), InfluxDB was chosen. For generating dashboards, Grafana was selected. All the developed virtual functions for monitoring, alerting, and coordinating were developed in Python.

#### 5.3.1. Temperature Distribution Visualization

**Figure 7:**
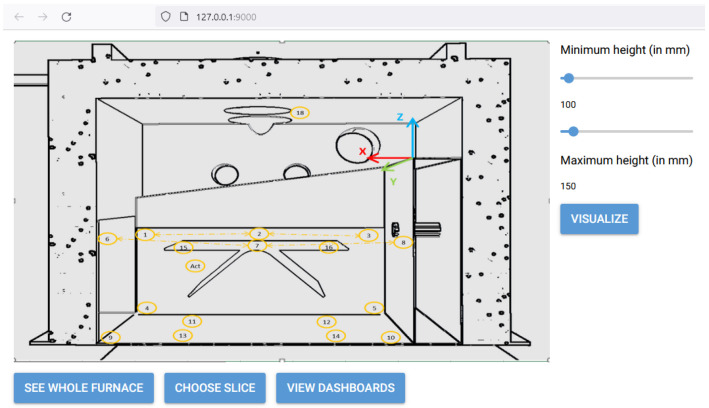
Initial page for furnace temperature visualization.

**Figure 8:**
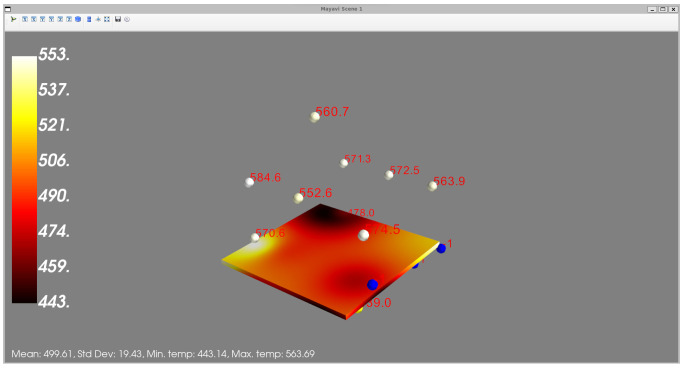
Visualization of selected slice.

**Figure 9:**
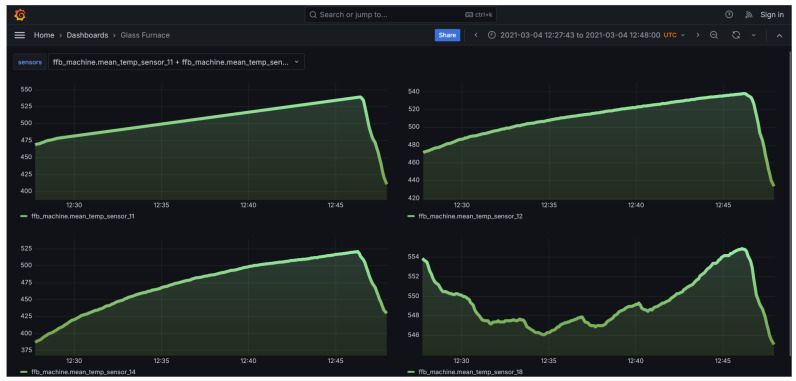
Grafana dashboard for thermal couple temperature visualization.

In Figure 7 and Figure 8, the developed User Interface (UI) from which the user can select to view the temperature distribution from the furnace used for glass bending is presented. Through the front-end, the users are given certain options from which they can choose either to view the entire furnace or a certain slice. In addition, by clicking the “View Dashboards” button, they are transferred to a Grafana dashboard page where they can see the evolution of the thermal couple values over time, as can be seen in Figure 9. Users can select from the options the subset of the thermal couples whose values they want to see plotted over time. Visualization of the temperature distribution is essential for the personnel handling the furnace because the mechanical properties of glass are strongly influenced by temperature, particularly in the range near or above the glass transition temperature [39]. As a result, the temperature distribution within the current furnace is a critical factor in the hot glass bending process. Currently, temperature gradients of up to 10 °C can be observed across the plane where glass workpieces are located. By utilizing furnace temperature visualization, the most uniform temperature location within the furnace can be identified. This enables the design of the glass bending process with the aim to improve the quality of the final product.

Besides the furnace, the devices used are thermal couples. The produced visualization is updated in real time from the thermal couples placed inside the furnace. The furnace VO retrieves the coordinates of the sensors and any disposition conditions from the cVO (i.e., the DT) orchestrating the operation, which have been retrieved from the KG. In order to produce the visualizations, a simple interpolation technique, called Inverse Distance Weighting (IDW) is employed. The premise of this method is that each sensor has more influencing power for points in the plane located close to it, with this influence diminishing for points located further away from it.

**Figure 10:**
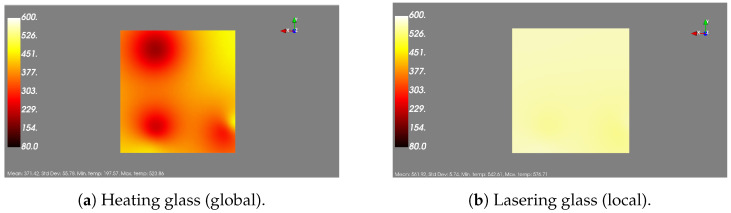
View of the same slice across two different time stamps. (**a**) 2021-03-04T12:21:05; (**b**) 2021-03-04T12:46:55.

The users can select the boundaries of the slice they want visualized along the Z-axis or they can select to view the entire furnace visualized (the outer walls). In order to produce the visualization, either for the whole furnace or for a slice, six surfaces are constructed, containing sampled points alongside each dimension. Then, for the sampled points, the temperature is interpolated based on the actual measurements of the thermal couples, which are also displayed in the figure alongside their measurements. The produced visualization is generated with the Mayavi Python library and it is interactive, meaning the user has the ability to rotate, zoom-in or out, etc. In addition to the visualization, the user can see the range of temperatures, the thermal couples displayed as spheres together with their measured values, and some simple statistics of the temperature metric (e.g., mean, standard deviation, etc.). The visualization is updated every 10s, which was selected as an acceptable interval that allows for avoiding computational overhead but still captures the changes in temperature. This visualization tool captures the changes in the temperature inside the furnace, as can bee seen in Figure 10, where the temperature distribution across two different time stamps corresponding to different steps of the procedure can bee seen. From the figure, it is easy to see that the heating of the furnace is reflected in the two screens, as the second one, which corresponds to a latter stage, also reports higher temperatures.

#### 5.3.2. Monitoring of Laser Glass Bending Process

One of the most important functionalities of the digital twin is the ability to monitor the entire business process. By accessing the KG, the DT is aware of the steps required and how each device that takes part in each step should be configured. This information is crucial because, on the one hand, it allows configuring the data flows between the different components (VOs) (i.e., what data to expect as input and when). On the other hand, this capability allows for increased control over the physical counterpart, as it can begin, pause, or terminate the operation of the equipment, provided that the necessary actuator IoT devices are embedded in the equipment.

**Figure 11:**
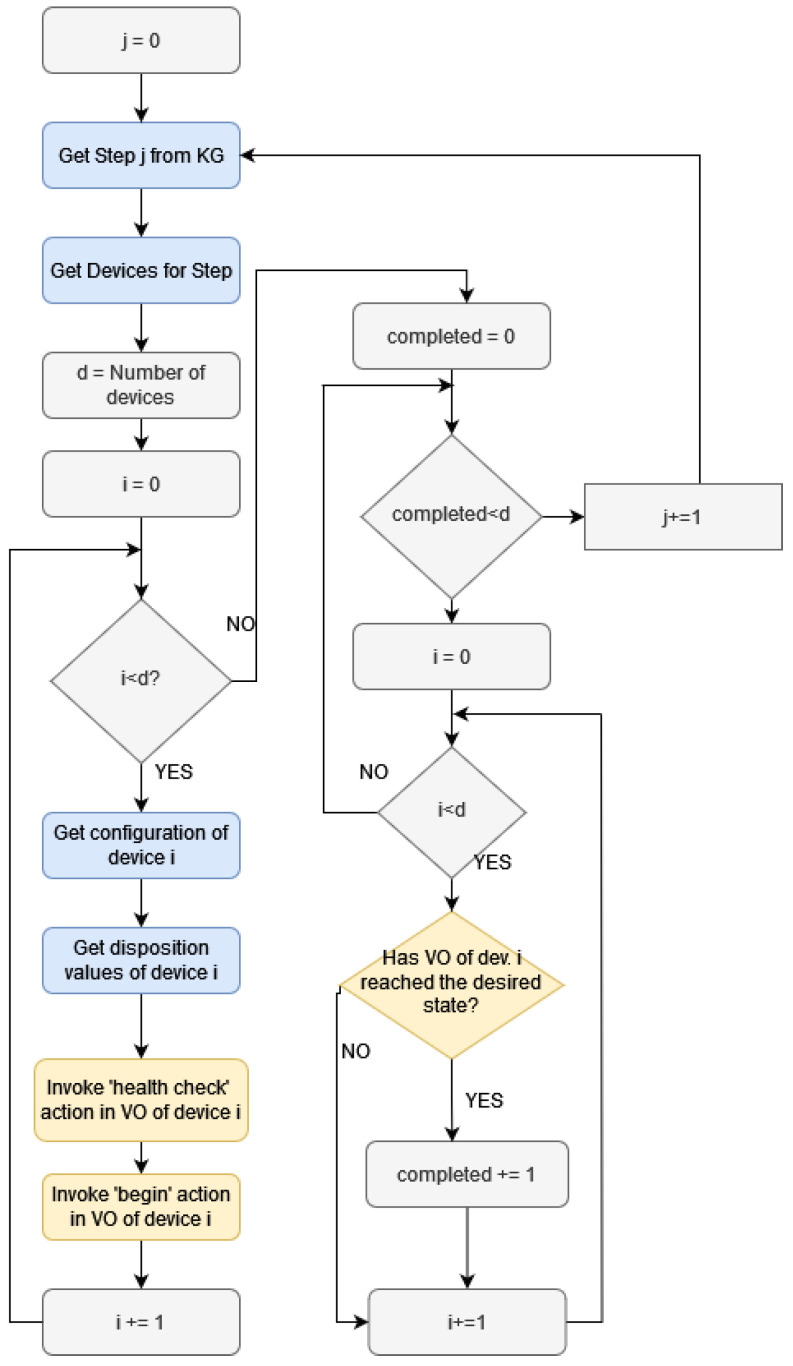
Monitor operation flowchart.

In Figure 11, a high-level flowchart of this functionality is displayed. The parts of the operation where the KG is employed are colored in blue and the parts where actions in the VOs are invoked by the DT are colored in yellow. For brevity, the details of each specific virtual function are omitted. In general, the operation begins with retrieving details on the specific step, the devices used, and their configurations from the KG. Then, each VO corresponding to the device begins two operations. The first one has to do with the necessary health checks in order to be able to monitor the operation of the real equipment (to ensure no “disposition” conditions are met). The next invoked operation is to begin the necessary virtual functions in each VO. Then, the DT polls each consumed VO in order to determine if it has completed the desired operation (e.g., reached a desired state). When all the VOs involved in a step have accomplished their tasks, the operation continues with the next step, until the IBP terminates.

**Figure 12:**
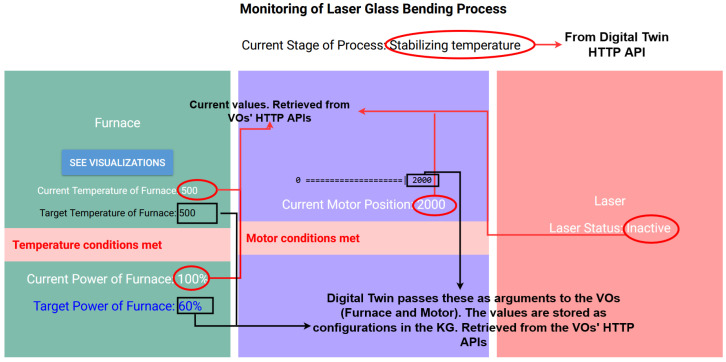
Webpage for monitoring the bending glass operation.

In the following figure (Figure 12), the developed front-end, allowing the user to monitor the overall process, is presented, with annotations that explain the origins of the various displayed values. The screen is divided into three parts, each one corresponding to a device. The metrics monitored by each VO and the corresponding target values for each step are displayed in the appropriate sections. The target values of the metrics (e.g., power and temperature) are stored in the KG in the respective device configuration nodes. The VOs are notified about these values from the digital twin (i.e., the bending glass cVO) after the latter queries the KG. The current values are retrieved from the current measurements of the VOs and reflect the condition of the manufacturing step.

## 6. Discussion

In this section, some interesting insights gained through the design and development of the proposed solution are discussed.

### 6.1. Suitability of the Solution to Handle Larger Use Cases

The proposed solution of combining a KG stored as a neo4j and developing the DT using the Nephele VO offers numerous benefits. First of all, for structured data such as those presented in this paper, using neo4j, a graph database, is faster than using a relational database [40]. Moreover, neo4j is a scalable database that allows for handling big data using sharding, i.e., dividing the single database in smaller databases (shards). In addition, the Nephele VO software stack is lightweight, allowing it to be deployed even in resource-constrained devices (e.g., Raspberry Pis). Moreover, the proposed architecture can combine operations in three tiers, namely, the physical, the edge, and the cloud. In this way, it can even be employed in resource-demanding applications that require significant computing power for data analysis.

### 6.2. Challenges and Limitations of Combining KGs and DTs

The proposed combination of KGs with DTs has been shown to work effectively especially in the specific setting presented in this paper, but, at the same time, one may identify emerging challenges and limitations in the more general case. Specifically, due to the more particular definition of a KG with respect to the considered application, at the moment, a holistic methodology to exploit the KG structure in an arbitrary DT is missing. A significant challenge, with a potential high pay-off, is to work towards a broader methodology capable of mapping a given KG structure to architectural components of a relevant DT structure. A second equally interesting challenge would be to explore the dynamic evolution of the combination, namely, the modification of the mapping in cases that additional functionalities and processes need to be included. Currently, such combinations seem to be more rigid; however, the descriptive power of the KG structure together with the aforementioned mapping methodology can potentially allow for the dynamic modification from the KG to the DT. Namely, if one can modify the KG structure to include processes or parameters, and a broader methodology for KG mapping to DT modules is available, it will be more straightforward to dynamically accommodate additions of processes and functionalities. Moreover, another challenge is to be able to provide digital twins, which can offer complex synchronization mechanism supporting in this way, manufacturing processes that involve numerous steps that should run in parallel. To accomplish this, an even more nuanced knowledge graph schema should be developed and an architecture containing numerous (c)VOs that communicate with the KG.

### 6.3. Benefits of the Proposed Solution for the Laser Glass Bending Scenario Presented

While the existing laser glass bending method, which does not utilize the digital twin with the knowledge graph approach, is capable of producing bent glasses with certain variations of geometric features, the manner of transferring the knowledge to bend glasses into different shapes (or even information for other types of glass materials) still remains a challenge. If the relationships between physical properties, process variables, and the sensor readings are not traceable, the efforts to generalize this laser bending technology to other applications will be significant. The combination of a DT and KG provides the solution to create extensive and executable process recipes to solve this problem. Capitalizing on the structured form of the KG, the users can access relationships among different physical properties of glass materials and equipment setups, while the DT can offer access to sensor readings obtained from the execution of different scenarios. All these can pave the way for creating simulations in a virtual environment that can help in deciding the optimal parameters to set in devices located in real facilities. In this way, costs will be reduced as less time will be spent performing experiments using the actual equipment.

## 7. Conclusions

In this work, we present a novel combination of a KG with a DT and its proof-of-concept application in a material manufacturing process. Initially, a novel KG schema is proposed, which is capable of incorporating knowledge relevant to the material industry. The coupling of this KG schema with the digital twin through the highly flexible software stack Nehpele VO allows for deployment of various algorithms. Due to its flexible schema, the KG stores information about the materials and the industrial business processes in which they partake. This allows for the development of a DT comprised of simpler software entities, which are referred to as Virtual Objects (VOs), whose parameters are dynamically configured and their operation is coordinated by the DT through suitable queries to the KG. This contribution is of broader value, as it can be exploited in other similar or relevant DT development attempts. This combination of tools (KG and DT) is beneficial since it allows a number of the DT’s functionalities to be enhanced, resulting in increased self-awareness. Finally, we present a tangible demonstration of the proposed approach in a real-world use case concerning a laser glass bending process.

Plans for future work include developing more advanced functionalities for the digital twin so that it can dynamically adjust its operation to any modification of the information stored in the knowledge graph. Moreover, it is our goal to design DTs that handle different parts of the business operations, encapsulating different IBPs with DTs that support an increased level of inter-communication.

## Figures and Tables

**Table 1:** Devices.

Device	“NEEDS”	“HAS_DISPOSITION”
Furnace	Heating power varies between 100, 60, and 0% according to step; Target temperature 500 °C	Heating rate < 5 K/min (20–400 °C) Heating rate < 2 K/min (400–600 °C) Heating rate < 1 K/min (>600 °C)
Thermal couples	Positions represented in (x,y,x) coordinates	Temperature values < 0 °C Temperature values > 1000 °C
Thermal camera	Filter: glass (20–900 °C)	Temperature of thermal camera housing > 70 °C
Motor	Moving from position 0 mm to position 2000 mm	No moving between 0 mm to position 2000 mm
Laser source	80% max. laser power	Laser power < 500 W
Laser scanner	The laser program is different according to the processed material	Laser speed > 10 m/s Laser jump speed > 50 m/s

**Table 2:** Steps.

Step	“SUPPORTED_BY”
Preheating furnace	Furnace, thermal couples, thermal camera
Glass enters furnace	Motor, furnace, thermal couples, thermal camera
Heating glass (global)	Furnace, thermal couples, thermal camera
Stabilizing temperature	Furnace, thermal couples, thermal camera
Lasering glass (local)	Laser scanner, laser source, furnace thermal couples, thermal camera
Glass exits furnace	Motor, furnace, thermal couples, thermal camera

**Table 3:** Laser scanner configuration per material and product.

Materials	Materials State	Laser Program	Products
Float Glass	Thickness 2.5 mm, edge polished	Laser speed = 3 m/s Number of laser lines = 10 Laser cycle = 100 Width between laser lines = 0.6 mm	Bent glass laminated outer automotive glass (radius 6 mm, bending angle 120°, thickness: 2.5 mm)
Float Glass	Thickness 5 mm, edge polished	Laser speed = 2 m/s Number of laser lines = 40 Laser cycle = 180 Width between laser lines = 0.5 mm	Bent glass single laboratory device door (radius 20 mm bending angle 90°, thickness: 5 mm)
Float Glass with low-E coating	Thickness 4 mm, edge rough	Laser speed = 3 m/s Number of laser lines = 10 Laser cycle = 120 Width between laser lines = 1.5 mm	Bent glass double glazing outer corner windows (radius 15 mm, bending angle 90°, thickness: 4 mm)

## Data Availability

No data is made available for the research work detailed in this manuscript.
